# Exploring resources and environmental carrying capacities at the county level: A case study of China’s Fengxian County

**DOI:** 10.1371/journal.pone.0225683

**Published:** 2019-12-02

**Authors:** Luyao Wei, Cheng Jin, Yuqi Lu

**Affiliations:** 1 School of Geographical Science, Nanjing Normal University, Nanjing, Jiangsu, China; 2 Jiangsu Center for Collaborative Innovation in Geographical Information Resource Development and Application, Nanjing, Jiangsu, China; 3 Key Laboratory of Virtual Geographic Environment Ministry of Education in Nanjing Normal University, Nanjing, Jiangsu, China; Institute for Advanced Sustainability Studies, GERMANY

## Abstract

The data regarding resources and environmental carrying capacities (RECC) are not only the basis for realizing regional sustainable development, but also the core links for major function-oriented zoning practices, which include specific partitioning schemes of spatial units with various geographical functions. Previously, relevant studies were mainly based on the evaluations of single factors. However, the realization of regional function-oriented spatial zoning practices based on comprehensive assessments of RECC has been neglected. This study presented an evaluation index system for RECC based on nine aspects, in accordance with the evaluation elements of the major function-oriented zoning programs which were in place and the characteristics of the study area. Then, by using subjective and objective comprehensive weighting methods, the basic elements were finally integrated, and an accurate spatial distribution pattern of the RECC in China’s Fengxian County was obtained. In addition, based on the construction of a three-dimensional spatial conceptual model, this study was able to finally obtain four specific types of functional partitions in the study areas, and proposed specific development proposals according to the different types of functional zoning from a systemic perspective. It was observed that the RECC had been decreasing from a central built-up area to the surrounding townships, and the spatial distribution patterns were distinctly scattered. In addition, the townships with smaller land scales had more obvious advantages in the RECC. However, fluctuating upward trends were observed after the lowest thresholds had been reached as for the medium or above medium scales. At the same time, in terms of the correlations between the population levels, and gross values and the RECC, fluctuating characteristics were observed. The correlations with the latter had presented S-shape curves and inverted U-shape curves, respectively. Finally, the optimized expansion zone located in the north-central region had taken the greatest percentage among functional zoning classifications, followed by the basic competitive zone in the southwestern section. However, the main construction zone accounted for the smallest proportion, at only 2.065%. Therefore, based on these results, it was concluded that there were certain fluctuating correlations between the RECC and total population levels, economic levels, and land scales. Moreover, the RECC evaluation results were found to gradually decrease after rising to the thresholds under the comprehensive effects of the various factors. This study combined the data of the conceptual model with the RECC evaluation results, in order to obtain a potential geographical functional zoning program for the study area. The results of this study are expected to provide a new analysis perspective for the scientific and sustainable development of small-scale geographic units. Moreover, on the basis of this study’s comprehensive evaluations of the RECC, the directions of regional development can be further clarified.

## Introduction

With the rapid development of China’s urbanization and economics, the constraints on resources and the environment have become increasingly tightened, and ecological problems are becoming gradually prominent. These issues tend to present conflicts between social and economic developmental needs to some extent [1]. Meanwhile, the development situations have not significantly improved, and it has become difficult to accommodate development potentials in some ecologically fragile areas [2]. The sustainability of China’s development is reflected in the sustainability of its resources and environmental carrying capacities (RECC), as well as government organization [3,4]. In another words, according to the dominant functions of the different regions in China, it is urgent that we clarify the supporting roles of resources and environmental conditions in certain spaces for the basic survival and development of the resident population. Recently, a great deal of attention has been given to the scientific and rational paths required to achieve sustainable social development by standardizing spatial orders and controlling developmental intensities. In addition, global resources and environmental carrying capacity (RECC) have been confirmed as having crucial impacts on the sound development of countries throughout the world.

In previous related studies, researchers have conducted various series of assessments of RECC around the world. Such investigations can be traced back to the origins of the research regarding carrying capacity, in which the French economist named Francois Quessnay explored the relationships between land productivity and economic wealth in 1758 [5]. Furthermore, since Malthus emphasized the restrictive effects after several years of food production on population growth, researchers have paid increasing attention to the application of carrying capacity data in economic and demographic studies [6]. For example, in 1921, biologists Parker and Burgess proposed a definitive concept of carrying capacity, which was subsequently used to measure the maximum number of individuals which could be sustained in a given area under certain environmental conditions [7]. These studies had later directly promoted relevant research in the field of land carrying capacities. Then, Odum presented a more accurate mathematical expression of the concept of carrying capacity in his book entitled *The Principles of Ecology* [8]. In the 1970s, the RECC of the Earth’s ecosystems began to be widely discussed. A series of representative research endeavors regarding ecological footprints and ecological appropriation were successively published in order to further improve the calculation methods for resource carrying capacities [9, 10]. From the perspectives of the quantitative relationships between the demands for natural resources of human activities’ and the Earth’s actual carrying capacity, the potentials for ecological balance and sustainable development among regions were explored. These explorations were then expanded and applied to the land resource carrying capacity research from the late 1980s to the early 20^th^ century. Moreover, corresponding concepts related to the carrying capacities of water resources, climatic resources, forest resources, mineral resource, tourism, transportation and other aspects, have been continuously proposed [11–14]. In fact, the concept of RECC is considered to be a total description of the maximum affordability threshold of regional systems in response to the external environment changes. It is known that a series of unbalanced load-carrying problems will result when certain limits are exceeded. Instead of a maximum population amount, the RECC represents the sizes of supported economies and the amounts of supplied resources and contained contaminants [15].

In addition to the evaluations of water, land, tourism, ecology, transportation, geology, and other single factors, RECC assessments can be gradually turned into multi-dimensional comprehensive investigations. The related research results have covered macro-, medium- and micro-scale levels, discussing the RECC of not only countries, but also urban agglomerations, regions, provinces, prefecture-level cities, municipal districts, county-level cities, and even disaster areas [16–18]. The findings of these previous studies offer a better understanding of the RECC in several ways. First, they have provided an understanding of the connotation and theoretical frameworks of RECC at different scale levels. Second, they have clarified both quantitative methods and mathematical models for comprehensive carrying capacity assessments, such as factor analysis methods, equation decision methods, state space methods, and analytic hierarchy process and entropy methods. In addition, they have made adopted such mathematical models [19] as coupling coordination models, three-dimensional transaction models, catastrophe progression models, driving-pressure-state-influence-response models, and so on. Finally, the previous RECC studies have promoted additional attention to be focused on the understanding of temporal-spatial dynamic evolution rules and the differentiation characteristics of different research units through RS, GIS, and other information processing technologies.

Although the existing studies provided relatively rich insight results, this study found that there have been minimal empirical research studies which have focused on RECC evolution at a county level. In particular, there has been a lack of spatial analyses encompassing different data types. The majority of the previous related studies were found to be based on single features or functional analyses, which tended to emphasize the assessments of the current development situations, while lacking comparative analysis results among various regional units. Moreover, the methods of index weighting were observed to be more subjective when evaluating the comprehensive carrying capacities. Therefore, the analysis results were not objectively representative. This study believed that comprehensive and accurate evaluations of RECC could only be obtained only by combing the socio-economic development and natural resource conditions of an area. However, the majority of the existing studies were related to developmental measurements in closed systems, without taking into account the flow of material and resources. On the other hand, it was found that statistical data were widely used to evaluate the relative amounts of RECC, while lacking absolute assessment combined with other types of data. In addition, the previous research had been limited to the comprehensive evaluations of RECC and the separate positioning of the main functions of specific areas. However, the correlations between the two had not been taken into account in the proposed targeted measures, as evidence and guidance for sustainable development.

In the present study, Fengxian County of China’s Jiangsu Province was selected as the research case. The goals of this study were to determine the core function locations of the different geographical units of the study area during the processes of spatial development and environmental protection by combining the acquired data with the geographic pattern changes of the land surfaces. There were determined to be four types of regions in the major function-oriented zoning scheme. The corresponding development strategic objectives were as follows: Optimized development; priority development; restricted development; and forbidden development [20]. In fact, the above-mentioned four types were characterized by different levels of RECC and development intensities. As one of the main or even paramount evidence factors for major function-oriented zoning evaluations, RECC has the ability to reflect the adaptability of resource-environmental and economic-social development. Therefore, it is beneficial to optimize resource allocations and resolve the contradictions between regional development situations and the actual demand levels [20]. Since RECC is not only the basis of regional development, but also provides important evidence for major function-oriented zoning, this study attempted to construct an evaluation index system of the RECC according to major function-oriented zoning practices. These included combined natural and human factors and social and environmental compound elements. As a result, nine indexes were chosen to assess the RECC, including land resource carrying capacities, water resource abundance, ecological vulnerability, ecological importance, flood disaster risks, soil environmental carrying capacities, transportation superiority degrees, population agglomeration degrees and economic development levels. Furthermore, this study adopted a method of comprehensive assignment by using both subjective and objective weights to obtain the final evaluation results. Then by constructing a three-dimensional spatial conceptual model of the RECC for the selected study area, taking townships as the basic evaluation units, a specific four types of functional partitions were obtained according to the assessment results of the RECC. Finally, different targeted developmental strategies were proposed. This research study attempted to provide a methodological reference and case study of the correlation analysis results between the RECC evaluation and the functional zoning process. It was found that by exploring the RECC at a county level, important contributions could be made in regard to promoting regional comprehensive development, as well deepening social and economic reforms.

The following section of this study demonstrates the characteristics of the study area, data sources, and adopted analytical methods. Moreover, the section on containing this study’s results and analysis findings provides the evaluations of the sub-indicators, along with a comprehensive integration evaluation of the RECC and its spatial differentiation law. On the basis of defining the main functional partitions using the RECC assessment results, the correlations between the RECC and the population levels, gross values, land scales, and so on, were revealed. The discussion and conclusion sections offer a summary of the entire analysis contents, as well as pointing out the current research deficiencies and possible prospects for future research.

## Material and methods

### Study area

**Fig 1:**
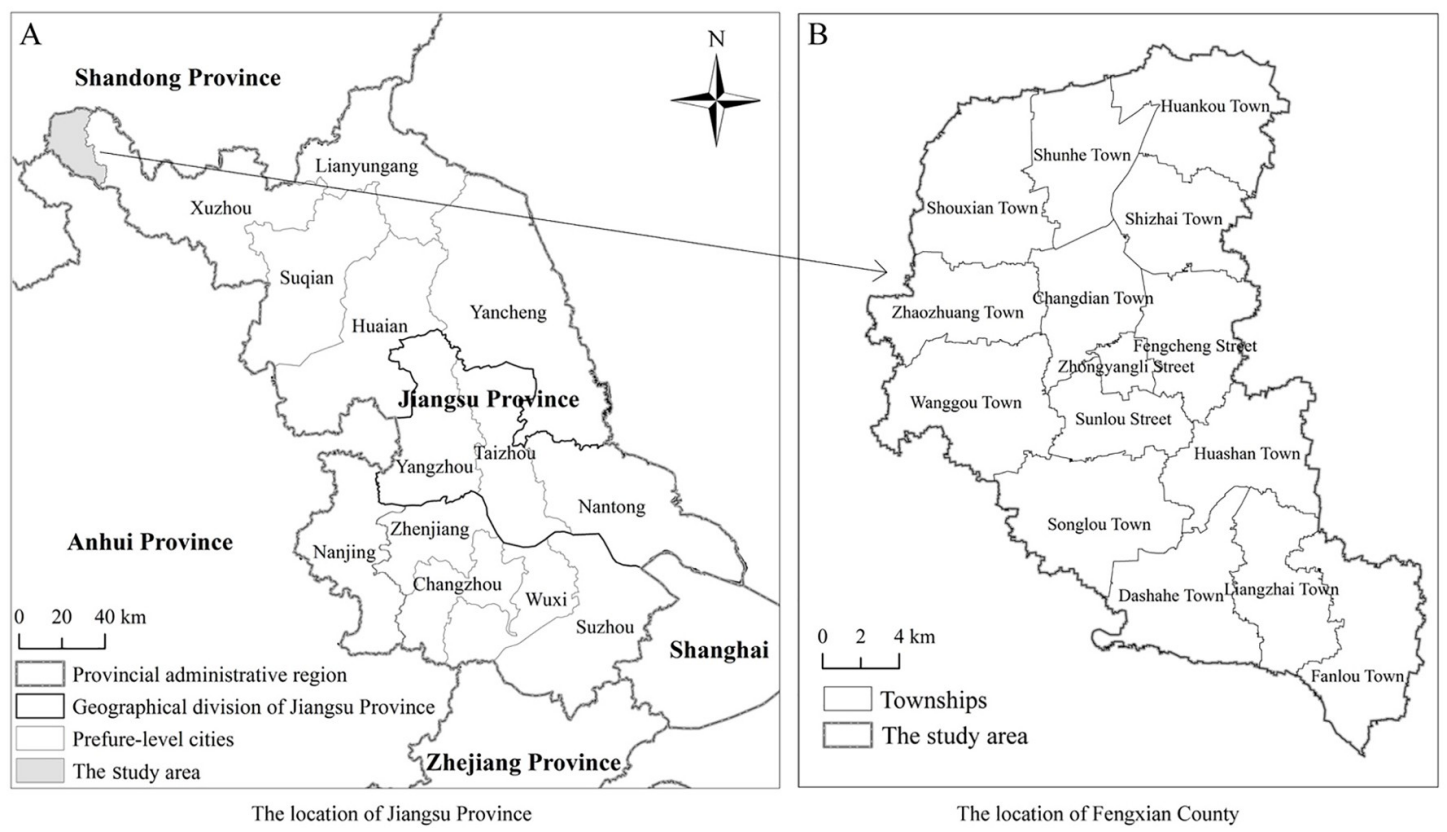
Geographical location map of the study area.

At the present time, the studies related to RECC have been focusing the majority of their attention on the analysis of RECC at the macro scale level. Meanwhile, RECC research at the medium- and micro-scales, such as districts, counties, and townships, are relatively scarce. As the basic research units and effective support for the implementations of spatial planning reforms, and the formulation of major function-oriented zoning schemes, RECC assessments at a county level have certain representative significance. Fengxian County is located in the northwestern section of Jiangsu Province, which is a subordinate of the Northern Jiangsu Province. Along with forming the junction between the four provinces of Jinagsu, Shandong, Henan and Anhui Province, Fengxian County is also the center of the Huaihai Economic Zone and the gateway city of the Xuzhou metropolitan area (Fig 1). The county consists of 15 townships characterized by high and flat terrain, as well a warm and humid year-round climate. There are also abundant water systems located within the county. The main rivers flow from north to south, with branches in an east-west direction. Among the major function-oriented zoning schemes, Fengxian County is listed as one of the restricted development areas. It is currently dominated by agricultural land, with cultivated land accounting for more than three quarters of the entire county’s land area. During a stage of urbanization transformation, appropriate directions for agricultural economic development, under the premise of ecological environmental protection, have obviously become important propositions for future scientific development projects in Fengxian County. These protection polices will also provide reference for the sustainable development of other similar small-scale regions of China even the entire world.

### Data sources

In this study, various spatial datasets and socio-economic statistical datasets were used as the data sources. As part of the investigation process, the corresponding basic geographic data from the 1:1000000 soil feature data set were obtained from the second soil census, as well as from the remote sensing image processing platform ENVI. In addition, the resources and environmental data cloud platform of the Institute of Geographic Sciences and Natural Resources Research (CAS) were utilized in this study. This study also projected data from 2016 provided by the Land and Resources Bureau, Transportation and Planning Bureau of Fengxian County. In regard to r the permanent resident population information at a township level in Fengxian County, the data was selected from the public security system. Moreover, relevant socio-economic and industrial development information was sourced from the *Fengxian Statistical Yearbook* of 2017.

### Research methods

#### Selection of the basic evaluation units

In the present study, for the purpose of avoiding possible "variable meta-problems" which may have resulted from misalignments between the administrative divisions and natural features [21], as well as to obtain the data of any regional differences in a more refined way, the study area was divided into grids of a certain size, which were regarded as basic evaluation units. This method was found to have a wide range of application, due to its effectiveness in dealing with huge amounts of data and weakening spatial information loss phenomena. Generally speaking, the grid sizes were determined under the conditions that the score gaps of each factor in the same grid were no more than 100/(*n*+1), while *n* represented the number of spatial functional units to be determined. In summary, this study chose to construct a grid unit measuring 50 m × 50 m.

#### Construction of the index system

This study selected nine indicators which were relatively consistent with the indicator system of the major function-oriented zoning based on the basic role of the RECC evaluations in the major function-oriented zoning process. These were separately evaluated for the carrying capacities of land resources, water resource abundance, ecological vulnerability, ecological importance, disaster risks, traffic dominance degrees, population concentration levels, and economy development levels [22, 23]. On this basis, by considering the effects of the intensities and directions of the actual soil environmental characteristics on the human social and economic activities in the study area, the index layer of soil environmental carrying capacity was also added as one of the determinants. Finally, by referencing the basic indicators of China’s major function-oriented zoning scheme, as well the data acquisition situation, this study successfully constructed a RECC evaluation index system which included nine subsystems and nineteen indicators (Table 1). As basic and indispensable resources for regional development and human survival, water and land resources play important pioneer roles in the evaluations of RECC. In particular, the perfect endowment of land and water resources in the study area can be seen to have significantly promoted regional economic development, and were found to have a certain degree of representativeness. At the same time, due to the increasing consumption of water and land resources during daily living activities, certain restrictions on the socio-economic development and RECC enhancement will inevitably result. At the same time, ecological vulnerability and ecological importance characterize the rationality of the structures and functions of regional ecosystems. It is possible to evaluate the material circulation and energy flow in RECC systems from the perspective of respecting the natural laws. In regard to assessing the possibilities of natural disasters occurring in an area, it is known that the higher the probability of disasters, the lower the results presented in RECC evaluations. In addition, the population agglomeration degrees, economic development levels, and transportation superiority, are considered to be basic indicators for characterizing human social and economic activities. These can be regarded as the basic conditions and opportunities for territorial space development in an area.

**Table 1:** Comprehensive evaluation index system of RECC.

Target layer	Index layer (impact)	Factor layer	General weight
**RECC**	**A**. Land resource carrying capacity (+)	Cultivated land area X_1_	0.283
		Reserved cultivated land area X_2_	0.191
		Economic value of cultivated land X_3_	0.316
		Construction land area X_4_	0.210
	**B**. Water resource abundance (+)	Available water resources X_5_	1.000
	**C**. Ecological vulnerability (-)	Terrain index X_6_	0.141
		Normalized difference vegetation index X_7_	0.296
		Species richness X_8_	0.332
		Land use degree X_9_	0.231
	**D**. Ecological Importance (-)	Distance to ecologically sensitive area X_10_	0.500
		Distance to river corridors X_11_	0.500
	**E**. Disaster risk (-)	Flood location index X_12_	1.000
	**F**. Soil environmental carrying capacity (+)	Soil organic matter content X_13_	0.338
		Surface soil texture X_14_	0.351
		Soil acidity X_15_	0.311
	**G**. Transportation superiority (+)	Road network density X_16_	0.713
		Time accessibility of critical traffic nodes X_17_	0.287
	**H**. Population agglomeration degree (+)	The amount of resident population X_18_	1.000
	**I**. Economic development level (+)	Economic industry density X_19_	1.000

Note: In the table, ‘+’ separately denotes the positive effects and ‘-’ indicates the negatives effect on RECC evaluation.

#### Calculation of the specific indicators

This study’s terrain index characterized the topographic attribute information by calculating the relationships between the slopes and the elevations of the grid units and average slope and elevation data of the study area. The regions with low elevations and slopes had small terrain and presented good natural conditions, as well a stronger RECC. The specific calculation formula used in this study was as follows:

$$I=\text{ln}\left[\left(\frac{\text{E}}{\prime \text{E}}+1\right)\right]\times \left[\left(\frac{\text{S}}{\prime \text{S}}+1\right)\right]$$ (1)

Where *I* represents the terrain index; *E* and ´*E* represent the average elevation of the grid unit and the overall elevation of the entire county, respectively; and *S* and ´*S* demonstrate the average slope of the grid unit and that of the entire study area, respectively.

The normalized differential vegetation index (NDVI) used in this study was a comprehensive reflection of the surface vegetation growth status and vegetation coverage. The negative values of the NDVI indicated that the ground cover was cloud, water, snow, and so on. Meanwhile, the positive values indicated that vegetation coverage existed on the ground surfaces. In addition, zero in the index represented that the ground objects were composed of rock or even barren in nature. The higher the values in the index, the better the vegetation grow on the land surfaces.

The species richness indirectly reflected the biological species abundance in the study area. The higher the biological index, the stronger the ecosystem vitality in the region. In the present study, by referring to relevant research results regarding the biological richness of county areas [24], the following formula was obtained:

$$\text{F}=(0.11\times {\text{S}}_{\text{cultivated}\;\text{land}}+0.35\times {\text{S}}_{\text{forestry}\;\text{land}}+0.30\times {\text{S}}_{\text{garden}}+0.21\times {\text{S}}_{\text{grassland}}+0.28\times {\text{S}}_{\text{water}\;\text{area}}+0.04\times {\text{S}}_{\text{construction}\;\text{land}}+0.01\times {\text{S}}_{\text{unused}\;\text{land}}/{\text{S}}_{\text{total}}$$ (2)

Where F indicates the biological richness; S_*i*_ represents the areas of the various land types, including cultivated land, forested land, gardens, grassland, water, construction land, and unused land, respectively; and S_total_ indicates the total area of the study region.

In the present study, the land use degree index reflected the natural attributes of the land itself during the land use processes. It was also a comprehensive reflection of the anthropogenic and natural environmental factors in the study area. To some extent, the land use degree index played an important role in reflecting the breadth and depth of the land use. This study referred to previous relevant research results [25, 26]in order to obtain the following formula:

$$\text{L}=\sum_{i=1}^{n}{\text{A}}_{i}\times {\text{C}}_{i}$$ (3)

Where A_*i*_ denotes the land use classification index of grade *i* within the study area; C_*i*_ is the proportion of the land use area of grade *i*; and *n* represents the number of land use degree classification (1≤*n*≤4). In accordance with the actual situation of the study area, the area was divided into unused land, grass and water, agricultural land and urban settlement land, with the assigned grading index (A_*i*_) of 1, 2, 3, and 4, respectively.

With consideration given to the dense water network and flood disaster occurrences in Fengxian County, this study chose the distance to ecological sensitive areas and river corridors as an index factor for the comprehensive evaluations of ecological importance. The calculation formula was as follows:

$${\text{P}}_{i}=\text{Max}({\text{ES}}_{i},{\text{R}}_{i})$$ (4)

Where P_*i*_ denotes the ecological importance index of the spatial units; ES_*i*_ demonstrates the ecological sensitive index; and R_*i*_ represents the river’s ecological importance index. Then, in accordance with the “ecological red line protection plan” and “13^th^ five-year plan for water conservancy and water affairs development planning”, this study determined the grading of the ecological sensitive areas and rivers within Fengxian County. Following the assignment of the different levels in the buffer analyses and specific investigations (Table 2), the ecological importance scores of the study area were successfully obtained by using a “maximum method” to superimpose the identified ecological factors.

**Table 2:** Discriminant factors and specific assignments of ecological importance.

Discriminant index	Grades	Classifications	Factor assignment
**Distances to the ecological sensitive areas**	First level of ecological sensitive areas	< 1,000 m: Core zone	100
		1,000 to 2,000 m: Buffer zone	80
	Second level of ecological sensitive areas	< 500 m: Core zone	80
		500 to 1,000 m: Buffer zone	60
**Distances to the river corridors**	Important rivers	< 200 m: Core zone	80
		200 to 500 m: Buffer zone	60
		5,000 to 2,000m: Affected zone	40
	Common rivers	< 100 m: Core zone	60
		100 to 200 m: Buffer zone	40

By using accessibility analysis technology, this study was able to calculate the time required for high-speed interchanges, railway stations, and ports in any area within the entire county, in order to characterize the accessibility of the key traffic nodes. Then, the impact scores of the key traffic nodes (including the above high-speed interchanges, railway stations, and ports) in Fengxian County were determined by the following formulas:

$${\text{S}}_{h,r,p}=\{\begin{array}{cc} 99\times \frac{\left(60-{\text{T}}_{h,r,p}\right)}{60}+1 & \left({\text{T}}_{h,r,p}<60\text{minutes}\right)\\ 1 & \left({\text{T}}_{h,r,p}<60\text{minutes}\right) \end{array}$$ (5)

Where S_*h*, *r*, *p*_ indicates the separate impact scores of the high-speed interchanges, railway stations, and ports; and T_*h*, *r*, *p*_ denotes the arriving times, respectively.

#### Weight factor determinations

The determination of the weight factors was of great significance to the evaluation process. In common cases, weight determinations may involve subjective or objective methods [27]. In order to reflect the RECC differences in basic evaluation units at a county level, and aiming at improving the credibility of the evaluation results, this study made use of a combination method for determining both the subjective and objective weight factors. It was found that only in this way, could the differential impacts of a single evaluation method reduce the number of the system’s comprehensive decision targets. First, the objective weights were calculated using a CRITC method, and the conflicts were measured using a comparison process and the correlations between specific indicators [28]. Then, by considering the influences of the measurement units, the factors were optimized by the coefficients of the variations. The specific formula was follows:

$$M_{j}=c_{j}\sum_{i=1}^{m}\left(1-r_{ij}\right)$$ (6)

Where *M*_*j*_ donates the amount of information contained in indicator *j*; in which the larger the *M*_*j*_ is, the more information the index *j* contained, and the greater the weight appeared; *c*_*j*_ is the coefficient of the variation of index *j*, in which $c_{j}=\sigma_{j}/\bar{x_{j}}$, $\bar{{\text{x}}_{j}}=\frac{\text{1}}{m}\sum_{i=1}^{m}{\text{x}}_{ij}$, and $\sigma_{j}=\frac{1}{n}\sum_{i=1}^{m}\left({\text{x}}_{ij}-{\text{x}}_{j}\right)$; and *r*_*ij*_ is the correlation coefficient between indicator *i* and *j*.

$${\text{r}}_{ij}=\sum_{i=1,j=1}^{i=m,j=n}\left({\text{x}}_{i}-\bar{{\text{x}}_{i}})({\text{x}}_{\text{j}}-\bar{{\text{x}}_{j}}\right)/\sqrt{\sum_{i=1}^{m}{\left({\text{x}}_{i}-\bar{{\text{x}}_{\text{i}}}\right)}^{2}\times \sum_{j=1}^{n}{\left({\text{x}}_{j}-\bar{{\text{x}}_{j}}\right)}^{2}}$$ (7)

Where the objective weight of factor *j* is:

$$\omega_{1j}=M_{j}/\sum_{j=1}^{n}M_{j}$$ (8)

Then, by combining the acquired results with those of the expert subjective weight recognition coefficient *ω*_2*j*_ for the total target, this study was able to construct a function according to the principle of minimum relative information entropy. The calculated the general weight is shown in Table 1. The formula could be written as follows:

$$\text{F}=\sum_{i=1}^{n}\omega_{j}\left(\text{ln}\;\omega_{j}-\text{ln}\;\omega_{1j}\right)+\sum_{i=1}^{n}\omega_{j}\left(\text{ln}\;\omega_{j}-\text{ln}\;\omega_{2j}\right)$$ (9)

Where $\sum_{i=\text{1}}^{n}\omega_{j}=\text{1},\omega_{j}>0$. Then, a Lagrange multiplier method was used to determine the optimal solution, and the weights of each factor could be obtained using the following formula:

$$\omega_{j}=\sqrt{\omega_{\text{1}j}\omega_{\text{2}j}}/\sum_{j=1}^{n}\sqrt{\omega_{\text{1}j}\omega_{\text{2}j}}$$ (10)

#### Integrated index evaluations

An integrated index evaluation method was adopted in this study. First, the index layers were measured using a multivariate factor analysis method. Then, the nine index layers were normalized according to their direction and nature in regard to the RECC.

In order to reflect the comparability and relevance between indicators, the positive indicators were calculated as follows:

$${\text{X}}_{j}^{\prime }=\frac{{\text{X}}_{j}-{\text{X}}_{jmin}}{{\text{X}}_{jmax}-{\text{X}}_{jmin}}\times 100$$ (11)

In terms of negative indicators, the following formula was used:

$${\text{X}}_{j}^{\prime }=\frac{{\text{X}}_{jmax}-{\text{X}}_{j}}{{\text{X}}_{jmax}-{\text{X}}_{jmin}}\times 100$$ (12)

Where ${\text{X}}_{j}^{\prime }$ indicates the normalized value of index *j*. Then, based on the comprehensive evaluation index system of the RECC of Fengxian County, this study successfully identified the attributes of nine secondary index layers. In regard to the appropriate quantitative index algorithm, it could be divided into two categories: a distributed algorithm and an integrated algorithm. The former included the determinants of the land resource carrying capacity; water resource abundance; soil environmental carrying capacity; population agglomeration degree; economic development level; and traffic dominance degree. These were then regarded as the leading indicators for development, due to the significant protection orientation of the population agglomeration degree, economic development level and traffic dominance degree. Meanwhile, the integrated algorithm included the three protective indicators of ecological vulnerability, ecological importance, and disaster risk, which are known to play fundamental roles in the comprehensive evaluations of the RECC. It was observed that the extents of the other secondary indicator roles, such as land resource carrying capacity, soil environmental carrying capacity, and water resource abundance, had not been clearly indicated. Therefore, when constructing this study’s comprehensive evaluation index, it was necessary to highlight the weights of the leading indicators. In contrast, the other indicators were mainly based on the theoretical attributes of their development and protection characteristics, with less participation. The formula of the comprehensive evaluation index was as follows:

$$\text{RECC}=k\times \sqrt{\frac{1}{3}\left({\text{G}}^{2}+{\text{H}}^{2}+{\text{I}}^{2}\right)}+\frac{\text{1}}{\text{max}\left(\text{C},\text{D},\text{E}\right)}$$ (13)

$$k=f\left\{\text{min}\left(\text{A},\text{B},\text{F}\right)|\begin{array}{c} \text{max(C},\text{D},\text{E)} \end{array}\right\}$$ (14)

Where *f* selects a function which matches the characteristics of the relationships between the resources/environment and the socio-economic development of the study area. Furthermore, the above formula guarantees that *k* takes a value between 0and1. In addition, the letters A to I refer to the corresponding secondary indicators shown in Table 1.

## Results and analyses

### Evaluation of the compositional factors

#### Land resource carrying capacity

**Fig 2:**
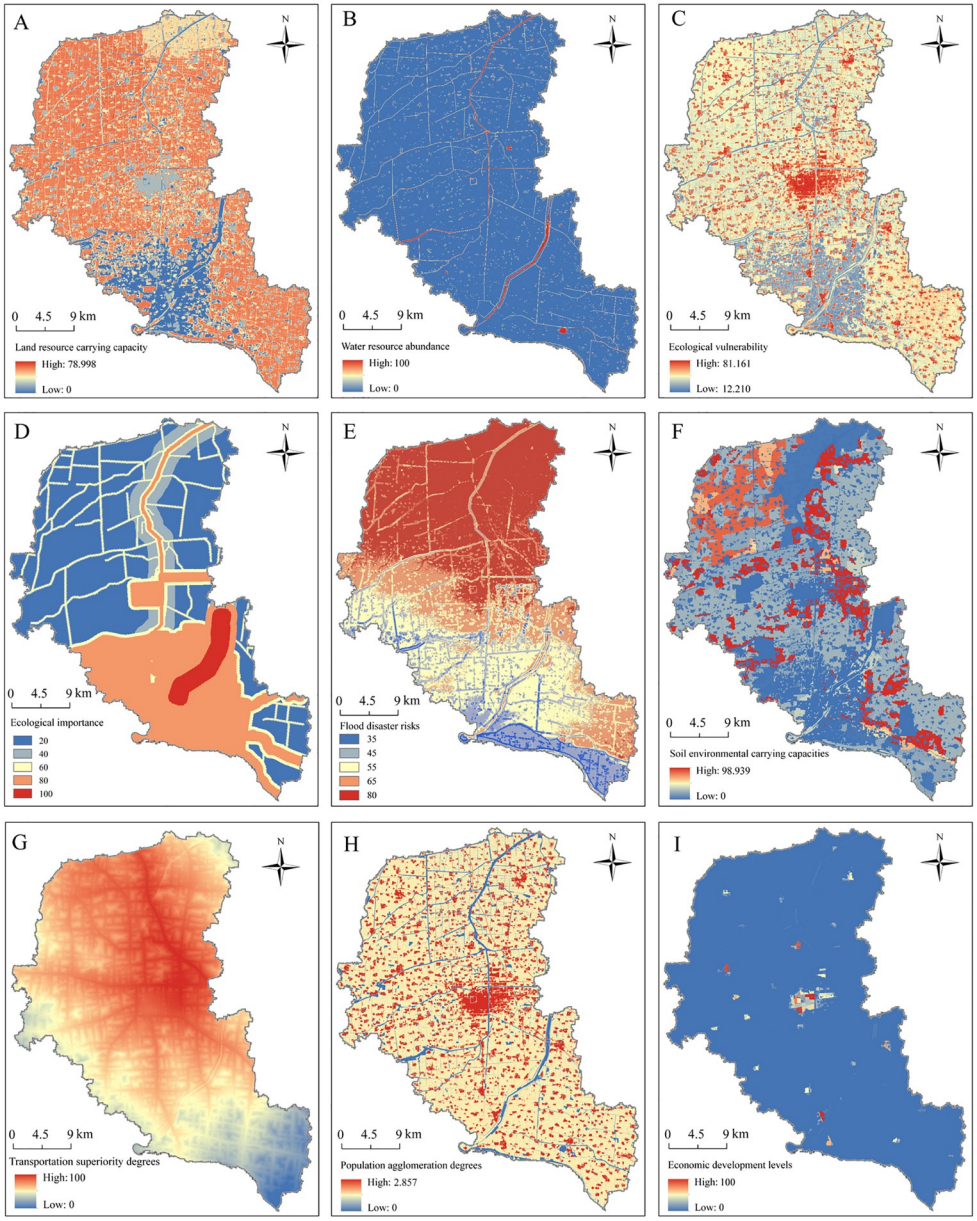
Spatial distributions of the compositional factors of the RECC.

The term land resource carrying capacity refers to the maximum carrying capacity of humans in regard to production and living on land resources under certain conditions of the population, economy, construction scales and ecological environment [29]. In regard to the resources and environment, not only do those factors depend on the land resources, but they also are known to have certain effects and influences on those resources. In the present study, the complex correlations among the land carrying capacity evaluation indexes and the social, economic, and environmental factors were considered. Subsequently, a subsystem was constructed for measuring the land carrying capacity of Fengxian County. This subsystem not only concluded cultivated land and construction land status areas, as well as the development potential of reserved cultivated land resources, but also the production capacity and basic quality of the cultivated land areas (economic values of the cultivated land). It was found to be noteworthy that the economic values of the cultivated land were characterized by the average economic coefficients of winter wheat and summer corn. It was found that as the economic coefficients of the cultivated land increased, the economic values of the cultivated land were promoted. In terms of the construction land areas, eight types of patches were extracted, including mining land, urban land, land designated for scenic facilities, ports and wharfs, highways, rural residential land, hydraulic construction land, and land areas containing railways. Then, using the weight assignments and normalization of the unified disposal, this study finally obtained the characteristics of the land resource carrying capacity, as detailed in Fig 2A. Generally speaking, the land capacity in the northern, southeastern, and western regions of Fengxian County were determined to be relatively high. Meanwhile, the overall level of the land carrying capacity in the southwestern area was observed to be lower.

#### Water resource abundance

The term water resource abundance refers to the amounts of water resources adjusted and utilized through engineering measures under technically feasible and economically reasonable conditions. The proportions of water area within a grid unit approximately represented the spatial difference in the available water resources within the county. In this study, water resource abundance was regarded as equal to the total area of the rivers, lakes, and ponds within a grid unit. Then, through the normalized handling of the source data, the characteristics of the water resource abundance in the study were determined, as detailed in Fig 2B. It was found that there was an abundance of water resources near the Fuxin River, Dasha River, and other drainage areas. However, there were fewer available water resources located in the central urban area.

#### Ecological vulnerability

Ecological vulnerability is the sensitive responses and self-recovery abilities of an ecosystem relative to the external disturbances at specific times and space scales. This results from the mutual influences of natural attributes and human economic behaviors [30, 31]. This study evaluated the ecological vulnerability degree in the study area from three perspectives: Ecological sensitivity, ecological resilience, and ecological pressure [32, 33], which was consistent with the theoretical basis of the ecological vulnerability assessments based on the connotation of ecosystem stability [34]. The self-regulation and resilience of an ecosystem under internal and external disturbances are closely related to the structural stability of the ecosystem. Therefore, apart from using the terrain index to reflect ecological sensitivity, this study also adopted the NDVI and biological richness in order to characterize the ecosystem resilience. The land use degree was used to demonstrate the ecological pressures. The results showed that the low-value areas of ecological vulnerability in Fengxian County were mainly concentrated in the river corridors and nearby areas. However, the areas with higher ecological vulnerability indexes were observed to be scattered and had mainly corresponded to rural residential areas. In addition, it was found that the ecological vulnerability levels of the built-up areas were the highest, as shown in Fig 2C.

#### Ecological importance

The assessment of ecological importance is based on the characteristics of ecosystem elements, which measures the output of ecosystem services [35]. According to the study’s evaluation results, we divided the study area into five classifications, namely extremely important area, comparatively important area, moderately important area, generally important area, and non-important area. These were then respectively assigned 100, 80, 60, 40 and 20 points (Fig 2D). The results show that the extremely important and comparatively important areas account for 33.1% of the total county, and they are mainly distributed in the drinking water protected areas, important wetlands and river trunks. As for the moderate important area, its spatial distribution is basically consistent with river network.

#### Flood disaster risks

Flood disaster is the main type of natural disaster in Fengxian County, which results a certain degree of inevitable casualties and economic losses. The topographic factor is closely related to the degree of flood hazard. The higher the terrain elevation is, the greater the likelihood that flood disasters will occur. It is easier to cause flood disasters with smaller terrain changes and lower slope values. Based on the grades of relative elevation standard deviation and slope, we constructed a discriminant matrix (Table 3), and consequently obtained the spatial differentiation of comprehensive topographic factors’ effect on flood disaster risk. Next, according to the specific flood disaster risk score, we divided the entire study area into five classifications, namely extremely dangerous area, comparatively dangerous area, moderately dangerous area, generally dangerous area and non-dangerous area. The different types were then assigned to 80, 65, 55, 45 and 35, by means of interval average value. Overall, the flood disaster risk in Fengxian County is shown to gradually decrease from north to south (Fig 2E).

**Table 3:** Grades of terrain elevation and their corresponding flood disaster risk.

Absolute elevation grades (m)	Elevation standard deviation grades (m)		
	First level [0, 0.50)	Second level [0, 1.26)	Third level [1.26, 7.14)
First level [28.46, 37.38)	90	80	70
Second level [37.38, 39.60)	80	70	60
Third level [39.60, 41.68)	70	60	50
Fourth level [41.68, 44.06)	60	50	40
Fifth level [44.06, 66.32)	50	40	30

#### Soil environmental carrying capacities

The soil environmental carrying capacity focuses on the measurement of scale, intensity, rate and composition of soil environment, without changing the soil environmental function. It emphasizes the extent of interaction between the soil environmental characteristics and the intensity, direction and scale of human socioeconomic activities [36]. In this study, based on dynamic and feasible principles, we selected the combination of three specific evaluation factors, namely soil organic matter content, surface soil texture and soil acidity and alkalinity, so as to further characterize the relative dispersed spatial distribution of soil environmental carrying capacities (Fig 2F).

#### Transportation superiority degrees

As an essential element of regional economic and social development, transportation facilities play an important supporting role in improving the regional accessibility and guiding industrial layout. The accessibility of high-speed interchanges in Fengxian County is generally strong, but the spatial distribution layout is not balanced. An obvious advantage can be seen in the northeastern area compared to the western and southern areas. About 85.04% of the areas can achieve high-speed interchange within 60 minutes, and 9.46% of the whole county can be reached within 40–45 minutes. The proportion of areas with high-speed interchange accessibility of greater than 60 minutes has also increased by 5.5%. By comparison, the accessibility of Fengxian Railway station is relatively poor, and the spatial distribution is equally uneven. A distribution of railway stations as the center and surrounding circular diffusion is shown. Specifically, the accessibility of railway stations in the northwestern and central regions is comparatively strong, while the northeastern, western and southern areas are weaker, due to their marginal locations. A total of 89.02% areas can access railway stations within 60 minutes, and the arriving time of the railway stations is concentrated within 20–40 minutes. However, accessibility of 25–30 minutes has the highest distribution frequency, with a proportion of 12.23%. As for the spatial distribution of port accessibility, a circular distribution is presented. The northern and central areas have stronger port accessibility, while the northeastern and southern regions are located far away from ports (Fig 2G).

#### Population agglomeration degrees

The population agglomeration degree is the most intuitive and concentrated reflection of the population spatial distribution pattern, and this is helpful for forming a useful supplement to the demographic data from different geographical scales and dimensions [37]. First, we adopted the deviation standardization method to dispose the population and land use data, so that all required data are mapped between 0–1. Next, we used standardized population data as dependent variable Y, and four types of land area data as independent variables X_1_, X_2_, X_3_, X_4,_ respectively. The intercept term in the linear regression model was set to zero [38]. According to the population dispersion coefficient (Table 4), we obtained the population of each 50 m × 50 m grid unit, as well the spatial discretization layout of population (Fig 2H). As consistent with actual life experience, the population is mostly concentrated in the developed area of the county.

**Table 4:** Linear regression between the population and types of land use factors.

Land use types	Natural reserved areas (X_1_)	Forested grassland, water areas (X_2_)	Cultivated land, gardens, agricultural facility land (X_3_)	Urban settlement land (X_4_)
**Correlation coefficient**	-0.205	0.028	0.342	0.603

#### Economic development levels

At present, Fengxian County is undergoing a key period of gradual transformation from agriculture-dominated to industry-dominated. The industrial structure of different villages and towns have a direct impact on regional development strategy adjustment. Next, according to the relationship between land use types and the development of primary, secondary and tertiary industry, we constructed a multiple regression model [39]. By using the method of regression analysis equation (Table 5), we determined the correlation between the primary industry output value (Y_1_) and the following land use types: garden (X_11_), paddy field (X_12_), dry land (X_13_), forested land(X_14_), reservoirs and potholes (X_15_), rivers and water surfaces (X_16_), irrigated land (X_17_), natural reserved areas (X_18_) and tidal flats (X_19_). Moreover, we also obtained the correlation between the total output value of secondary and tertiary industries (Y_2_) and the following land use types: urban land (X_21_), rural residential land (X_22_) and other construction land (X_23_), the latter of which includes mining land, ports and wharfs, highways, rural road, irrigation and water conservancy land, agricultural facility land and hydraulic construction land.

**Table 5:** Linear regression between the economic development levels and land use types.

Type	Linear regression equation	Correlation coefficient
Economic density of the primary industry	Y_1_ = 7.43825X_11_+9.92326X_12_+5.55844X_13_+55.14572X_14_-106.22108X_15_−4.12414X_16_ +662.65415X_17_−0.40912X_18_+84.63683 X_19_	0.985
Economic densities of the secondary and tertiary industries	Y_2_ = 712.00265 X_21_-43.50090 X_22_+137.44720 X_23_	0.905

After completing the significance test, we set the constant term as zero, and spatialized the economic density of the primary, secondary and tertiary industries. The development of the primary industry in Fengxian County has a significant positive correlation with garden land, paddy field, dry land, forestry land and tidal flats. Among these, forested land, tidal flats and paddy field are the most relevant categories. However, reservoirs, potholes, rivers and water surfaces, as well natural reserved areas, all play a negative feedback role on the output value growth of the primary industry. In addition, the second and third industries have strong positive correlation effects with the urban land and other construction land. Rural residential land does not have a positive impact on the development of the secondary and tertiary industries, due to its own strong productive function. On the premise of determining the correlation between industrial development and land use classifications, we superimposed a grid with 50 m × 50 m accuracy with the land use data, and obtained the ultimate result of economic density (Fig 2I). Generally speaking, the economic development of the developed areas and the vicinity of township centers is relatively high, which is consistent with the conventional recognition.

### Comprehensive evaluation of RECC

**Fig 3:**
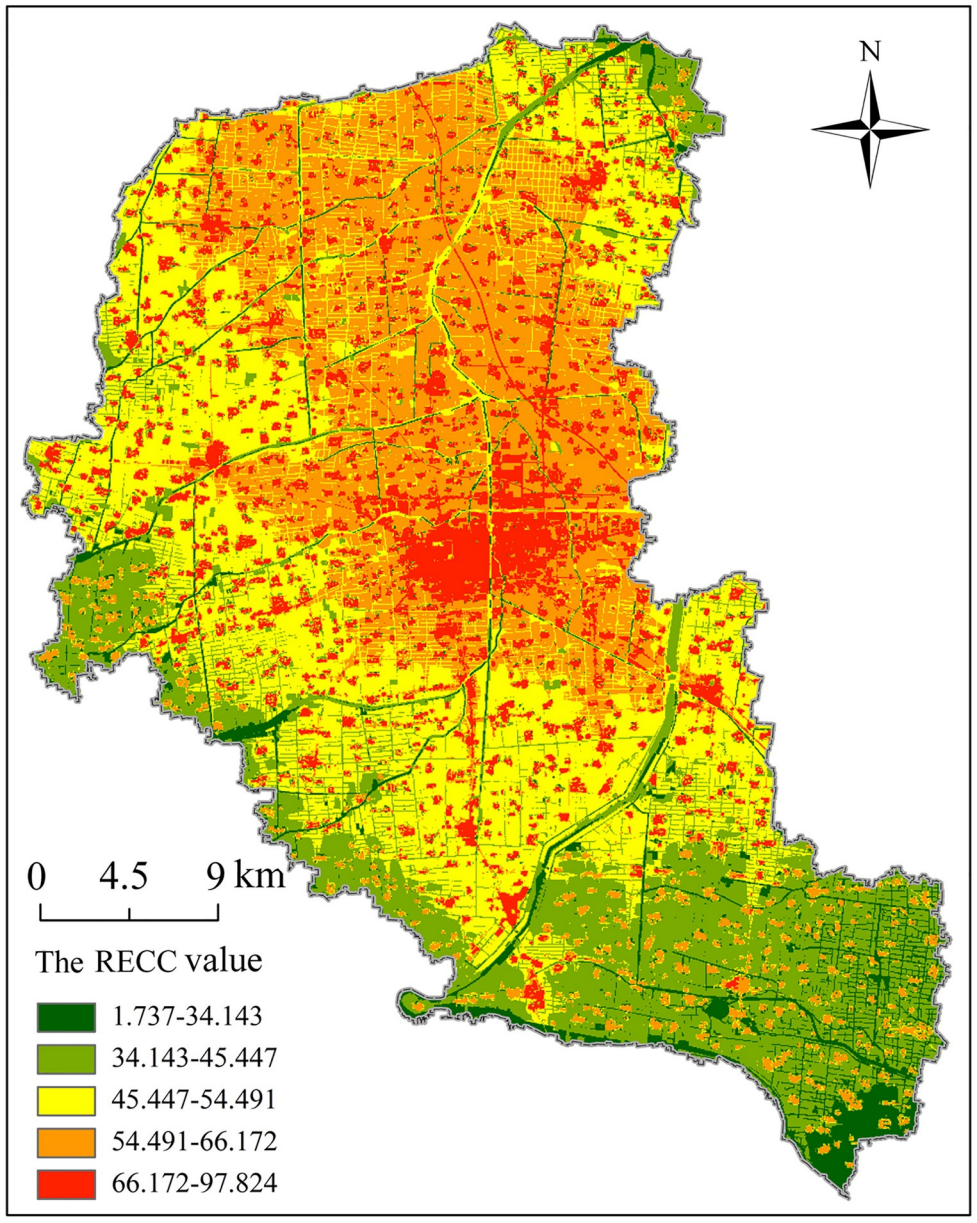
Spatial distributions of the comprehensive RECC.

**Fig 4:**
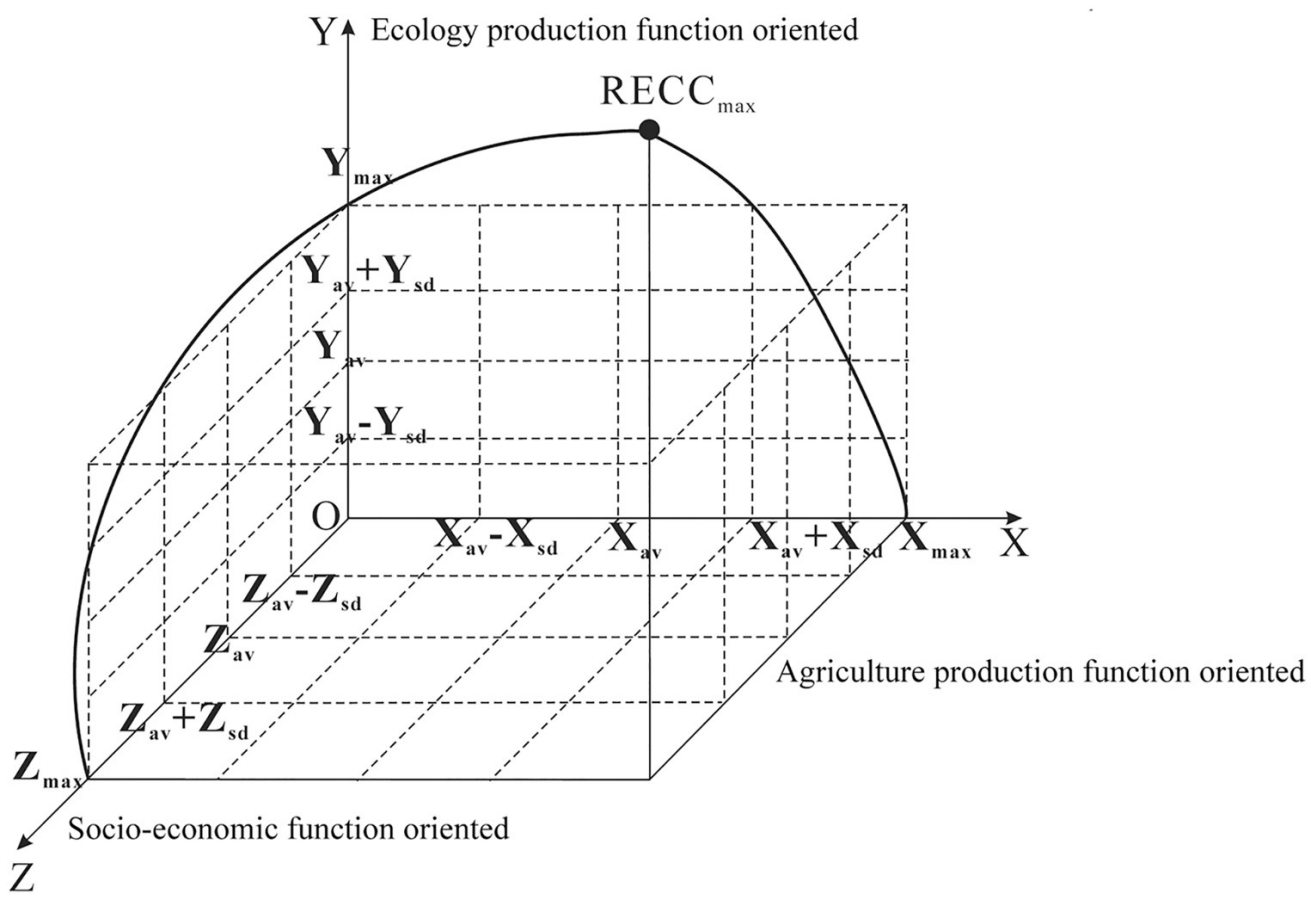
Three-dimensional spatial conceptual model of the RECC.

According to the impact direction and properties, we normalized the nine secondary index indicators, and then, using the adopting comprehensive integration evaluation method, we obtained the evaluation of RECC. The RECC in the central built-up area of Fengxian County is the highest, while it still needs to be further explored in the southeastern region (Fig 3). Overall, the RECC in Fengxian County basically presents a stepped distribution pattern, which gradually decreases from the center to the edges. Next we used a three-dimensional spatial conceptual model to visually reflect the relationship between the RECC and the functional zoning (Fig 4). By identifying the node classification and attribute characteristics, we partitioned the main functions embodied by each element into numerous categories. The elements of the three-dimensional spatial conceptual model of the RECC were mapped to the X axis, Y axis and Z axis, which respectively corresponded to the three main functions of agriculture produce function oriented, socio-economic function oriented and ecology produce function oriented partitions. Referring to relevant research [40], we determined the number of nodes in three-dimensional spatial model on the basis of the functional relationship between the average value and standard deviation of the RECC, then we obtained four interval ranges (Table 6). According to the distance from the nodes to the origin point O in the three-dimensional spatial conceptual model, we acquired the attribute values of each mode. The farther the distance to the origin is, the more representative the main function is, and vice versa, i.e. the lower the level is, the more difficult it is to fully reflect the functional characteristics of the basic elements. In the conceptual model, the three-dimensional space unit forms a dominant function zoning via different combination forms and classification standards of major functions. The RECC of evaluation units will gradually decrease after rising to the threshold under the comprehensive effect of numerous factors.

**Table 6:** Main functional partition led by the RECC evaluation results.

Advantage partition	Main oriented function	Conceptual model coordinates
Areas with absolute predominance (Main construction zone)	Agriculture production Ecology production Socio-economic	x∈[X_av_+ X_sd_, X_max_], y≥Y_min_, z≤Z_av_+Z_sd_ x≥X_min_, y∈[Y_av_+ Y_sd_, Y_max_], z≤Z_av_+Z_sd_ x≥X_min_, y≤Y_av_+Y_sd,_ z∈[Z_av_+ Z_sd_, Z_max_]
Areas with comparative advantage (Optimized expansion zone)	Agriculture production Ecology production Socio-economic	x∈[X_av_, X_av_+ X_sd_], y≥Y_min,_ z≤Z_av_ x≥X_min,_ y∈[Y_av_, Y_av_+ Y_sd_], z≤Z_av_ x≥X_min,_ y≤Y_av,_ z∈[Z_av_, Z_av_+ Z_sd_]
Basic competitive zone	-	[X_av_-X_sd_, Y_av_-Y_sd_, Z_av_-Z_sd_]≤x, y, z≤[X_av_, Y_av_, Z_av_]
Potential excavation zone	-	[X_min_, Y_min_, Z_min_]≤x, y, z≤[X_av_-X_sd_, Y_av_-Y_sd_, Z_av_-Z_sd_]

Note: In the tabel, x, y and z respectively represent the levels of the agricuture production function oriented areas, ecology production function oriented areas, and social-economic function oriented areas.

**Fig 5:**
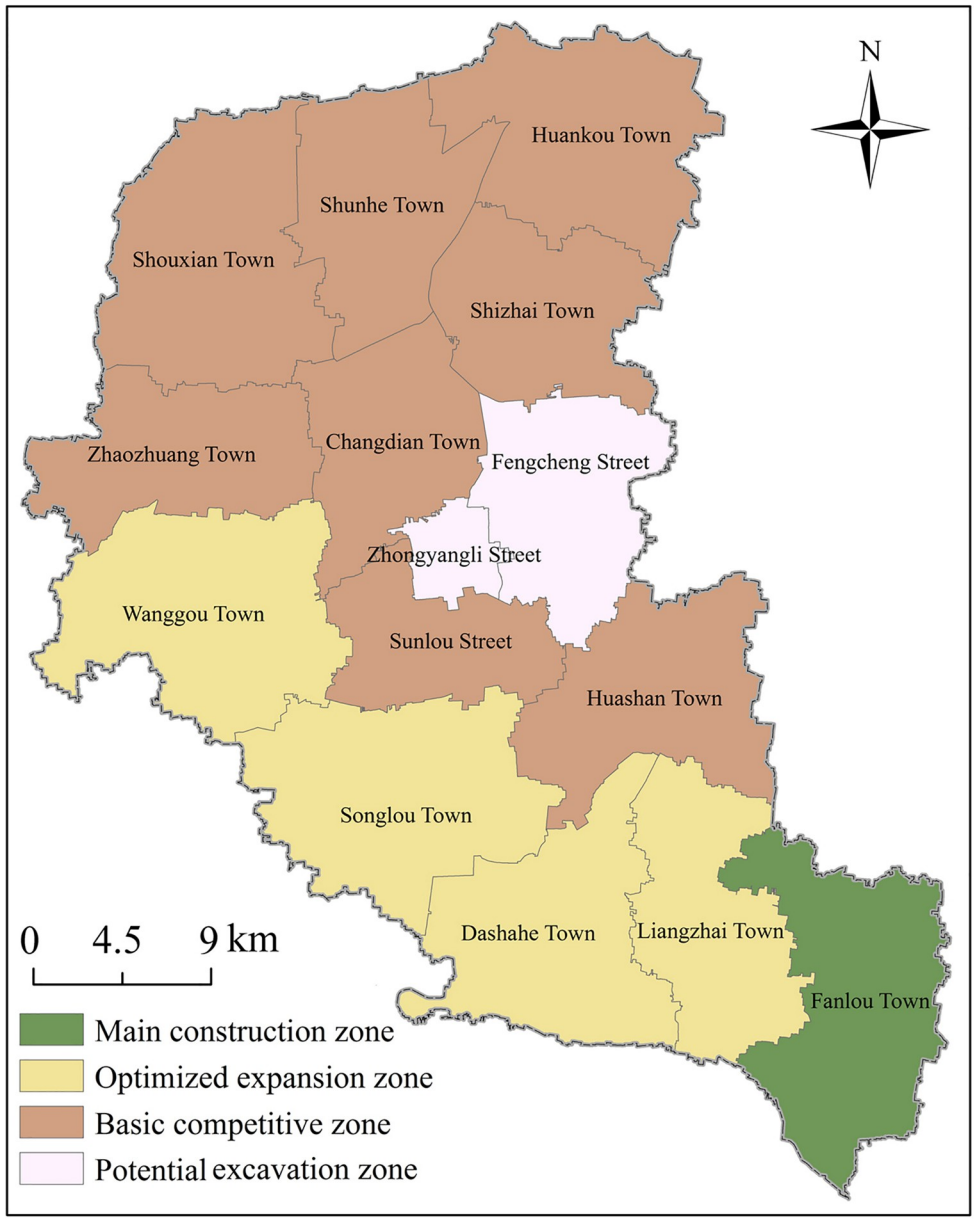
Evaluation of the RECC at the township level.

**Fig 6:**
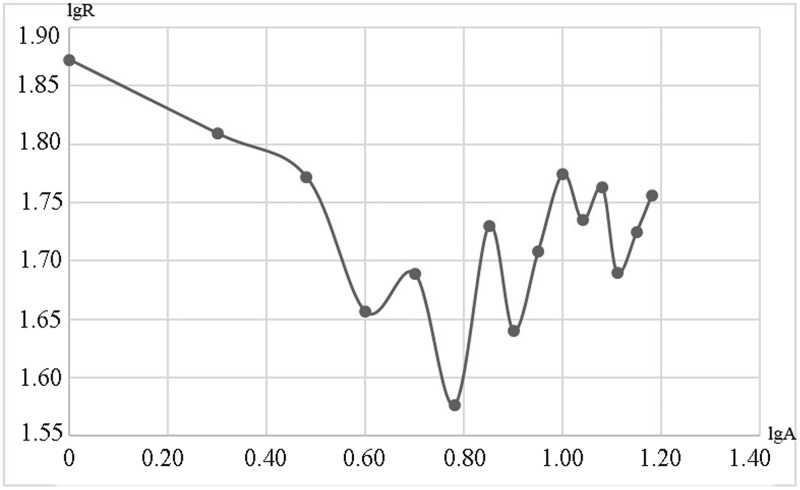
Rank-size analysis results between the RECC and land scales.

According to the statistics of the RECC in sub-towns, we obtained the evaluation range of RECC and the ranking divisions in Fengxian County (Fig 5). The maximum evaluation score of the RECC in the county as a whole is 74.557, while the minimum is 37.722, which respectively correspond to Fanlou Town in the most southeastern area and Zhongyangli Street in the central area. We analyzed the relationship between RECC and land scale by rank-size law. The results show that the townships with smaller land area have more significant advantages in RECC, while the RECC of towns with medium or higher scales show a fluctuating upward trend after the lowest value (Fig 6). Referring to the above-mentioned major function-oriented zoning guidelines and actual situation of Fengxian County, we formed a territorial space development pattern. As can be seen, Fengxian can be further divided into four types of areas, namely main construction zone, optimized expansion zone, basic competitive zone and potential excavation zone with gradually decreasing RECC. Among these four types, sub-towns with the largest proportion are the optimized expansion zone, followed by basic competitive zone. However, the latter accounts for less than half of the former. As for the remaining 8.343% of the county, this consists of potential excavation zone and main construction zone, with the former being as much as three times as large as the latter. The reason for the formation of current spatial pattern is that, the development potential still needs to be excavated due to the combination of high flood risk and poor traffic accessibility conditions in the southeastern region. In addition, the overall effect of natural resource endowment and socio-economic conditions leads to the formation of basic competitive zone. Moderate ecological vulnerability, disaster risk and traffic dominance together lead to basic competitive zone formation. Under the conditions of agglomeration population, strong economic development level and convenient transportation, an obvious advantage of the RECC in the main construction zone becomes evident. Through the radiation diffusion effect, the sub-towns and streets around the built-up area become the potential excavation zone.

**Fig 7:**
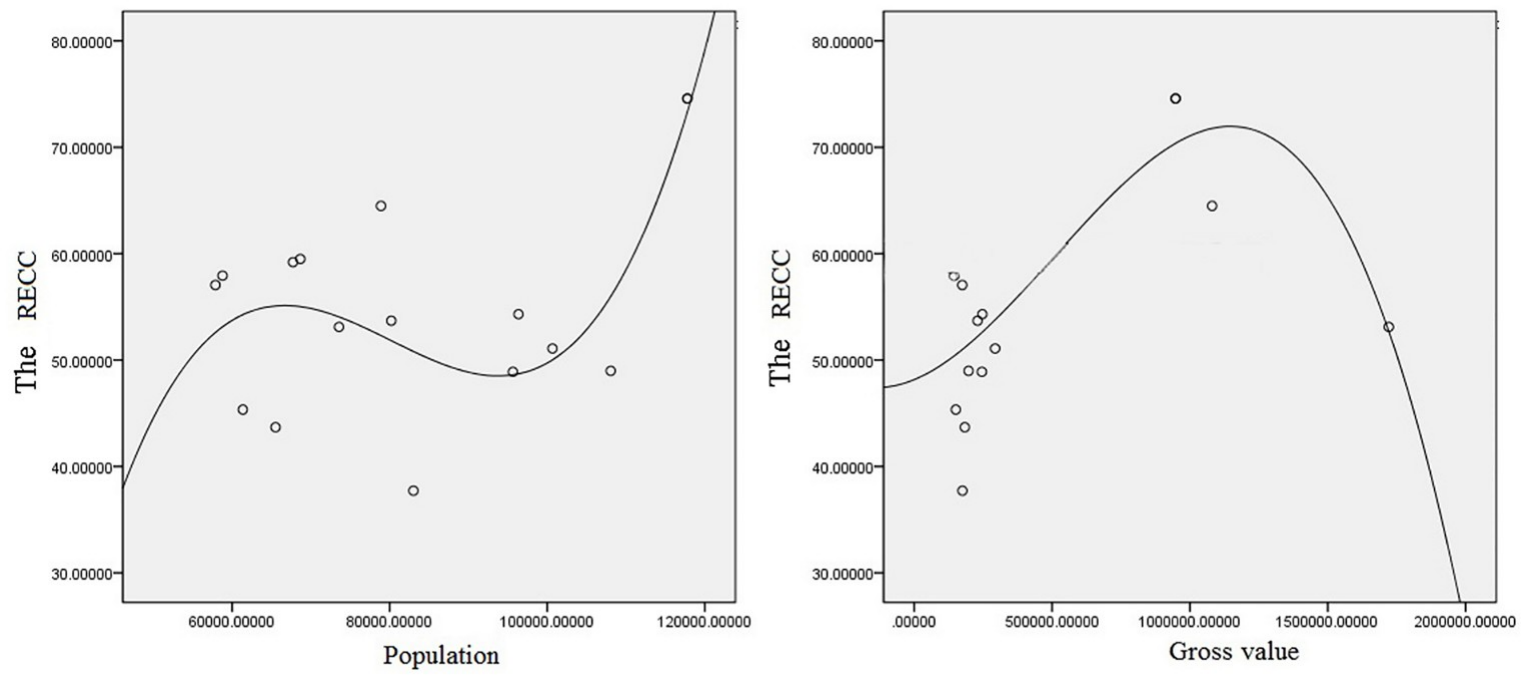
Correlations between the population levels and gross value and RECC.

In terms of the correlation between population, gross value and RECC, a fluctuant characteristic can be observed (Fig 7). In particular, the relationship between the population scale and RECC presents an S-curve shape. With the increase of population scale, the value of RECC first increases, then decreases, and finally continues to improve. The most concentrated population area has the largest RECC value. On the other hand, an inverted U-shaped correlation can be observed between the gross value and RECC in Fengxian County. Different from a balanced and scattered population distribution, the industrial economic development level of townships or streets in Fengxian County is relatively centralized and consistent. While only the developed areas of Zhongyangli Street and Fengcheng Street maintain a high level of industrial economic output, and they are still equipped with a high RECC value. Despite the fact that Zhaozhuang Town has improved its industrial development level, its RECC is slightly decreased compared with the former two.

### Functional zoning based on the RECC evaluation results

The assessment of the RECC at the county level is an important basis and foundation for optimizing spatial layout. Taking this as the basic premise, and combining this with the natural resources endowment, ecological environment and social-economic conditions of Fengxian County, we then further analyzed the main characteristics of the function partitions, and proposed relevant policy recommendations.

Main construction zone is concentrated in the main developed area of Fengxian County with the greatest concentrated population and positive economic activities, which covers Zhongyangli Street and Fengcheng Street. It also plays a core role in radiating the development of other towns and streets. The development density is very high, while the RECC value tends to weaken. In the future, in order to create a region with rapid economic development, as well a harmonious and livable environment, we must strengthen environmental governance, optimize industrial restructuring, and accelerate the upgrading of public infrastructure services.

On the basis of the radiation effect of developed areas, the optimized expansion zone has been gradually developed due to its transportation advantages and abundant resources conditions. It has become an inherited development zone with a strong RECC. In the future, this region must give full play to the distinctive advantages of industries while protecting the ecological environment, and by combining with resources and location advantages, as well industrial linkages with surrounding areas.

Wanggou Town, Songlou Town, Dashahe Town and Liangzhai Town in the southwestern area are considered to be the basic competitive zone. This region is rich in water resources and agricultural utilization conditions. From the perspective of optimizing land spatial distribution, we should strengthen disaster prevention and control measures. Aside from this, soil environmental quality must be monitored and guaranteed by means of modern production technology, so as to ameliorate and improve land resource efficiency.

Potential excavation zone refers to a region with weak RECC, poor habitability of ecological environment and transportation convenience. More importantly, the large-scale agglomeration economy and population conditions in this region still require strengthening, due to natural disasters. We must also give greater consideration to creating a model of characteristic ecological settlements, while striving to achieve all-round development of ecology, resources, society and economy.

## Discussion

As one of China’s top 100 counties with the greatest development potential, it appears that the agriculture of Fengxian County has maintained a good development tendency for many years, aiming at building up an excellent agricultural county among Jiangsu Province, and even the entire country. On the other hand, Fengxian County also has strong resource endowment and ecological environment, such as fertile land, and rich productions. However, in the stage of urbanization transition, as a restricted development area in the function-oriented spatial zoning, the integration and conflict between social and economic development and natural resources and environmental conditions of Fengxian County have become increasingly prominent. The resources and environmental conditions within a certain period of time and geographical spaces are limited, and the functional guarantee degree and scale bearing capacity of human activities have also reached the maximum threshold. When the pressure of human social and economic activities reaches or even exceeds this threshold, irreversible damage will be caused to the ecological environment, as well the sustainable development of economy and society. Under the dual rational adjustment of natural systems and human activities, the RECC gradually departs from the maximum threshold, then maintains a steady situation. Therefore, it is of great significance to evaluate the RECC so as to clarify its functional zoning and development direction. In brief, balancing the relationship between economic development and ecological environmental protection in Fegnxian County has become an important issue. To a certain extent, this determines whether the country will be able to achieve scientific and sustainable development.

Although researches regarding RECC have made some progress in urban agglomerations, prefecture-level cities, water resources, land resources, tourism resources and other aspects, how to evaluate the RECC at the county level remains to be elucidated. The present study used a small-scale county unit as the analysis object, which has extensive operability and practical application value. Different from the common subjective weighting methods of statistical data, we adopted subjective and objective comprehensive weights to analyze the RECC of refined spatial grid units. On the premise that RECC evaluation is an important foundation for major function-oriented zoning, we constructed a three-dimensional spatial conceptual model. Finally, we obtained specific function partitions, including main construction zone, optimized expansion zone, basic competitive zone and potentiality excavation zone. In addition, we proposed the correlating development suggestions of each functional zoning. The research results show that the RECC in the northern section of Fengxian County is significantly higher than that in the southern area, and is the strongest in the developed area. The RECC value has a strong correlation with economic and social conditions, but it is not necessarily negative. In terms of the study area, the correlation among population, gross value and RECC shows a fluctuant characteristic.

Compared with other related studies [18, 19], this paper attempts to measure the RECC based on more refined methods and a smaller scale perspective, and takes reference to its relationship with the major function-oriented zoning, so as to build a quantitative model and further clarify the direction of regional development. In fact, the current research on RECC assessment mostly reveals the interaction between the pressure exerted by human social-economic activities and undertaken by natural resources and environment [1, 2]. However, it does not effectively answer the question regarding the carrying potentiality. It is suggested that we must better combine the evaluation of ability, pressure and potential of the RECC with policy factors, so as to provide support and reference for functional zoning and spatial planning decisions. At the same time, in view of the subjective initiative of human activities, it is difficult to ignore the proposition of how to determine the threshold and overload situation of non-linear changes. In addition, considering the short-term carrying situation of resources and environment, we can further discuss it in the long term period, thereby providing support for realizing the predictability of spatial planning. In terms of technical methods, comprehensive researches on the RECC are relatively weak, which mostly focus on static states, and lack dynamic measurements of non-closed systems with substances and energy cycles. As for practical application, we may consider comparing the RECC of different natural geographical environments, and exploring the relationship between topographical features, economic development level, etc. and the RECC. However, some questions remain, such as how can we scientifically identify the key limiting factors of the RECC combined with the short-board effect? How can we quantify the lubrication effect of management technology progress on the RECC? And how can we thoroughly explore the formation mechanism and evolution law of the RECC? These theoretical issues must also be further explored. In this study the realization of the analysis framework is based on the characteristics of the study area, but the evaluation of RECC involves a greater number of judging elements. Therefore, it is necessary to establish different evaluation subsystems according to the specific conditions of the study area, and to enhance the universality of analysis framework, while gradually improving the evaluation system.

## Conclusions

The term RECC refers to the ability of regional resources and environment system to withstand various social and economic activities within a certain period and region, under the conditions that the regional resource structure meets the requirements of sustainable development, and that it maintains a steady-state effect. According to the basic role of the RECC in the major function-oriented zoning, combining with the characteristics of study area, nine secondary indicators were synthetically integrated by both subjective and objective comprehensive weighting method. Specifically, the criteria are land resource carrying capacity, water resource abundance, ecological vulnerability, ecological importance, flood disaster risks, soil environmental carrying capacities, transportation superiority degrees and economic development levels. Finally, we obtained the spatial distribution pattern of the RECC in Fengxian County of Jiangsu Province, China. The RECC value is relative scattered, and tends to weaken from the developed area to the surrounding areas. On this basis, according to the relationship between the RECC and the main functional partition, we classified the entire county into four functional partitions, namely the main construction zone, optimized expansion zone, basic competitive zone, and potential excavation zone. Among these, the optimized expansion zone accounts for the highest proportion, at about 63.391%, while the main construction zone accounts for the smallest proportion of the total county, and is mainly concentrated in the developed area. Through the rank-size law, we determined the correlation between the land scale and RECC. Next, we found that in the townships, the smaller the land scale was, the higher the RECC would be. The population and RECC of Fengxian County show a fluctuating correlation with an S-shaped curve, while the economic output value exhibits an obvious inverted U-shaped relationship with the RECC. Based on the natural resource conditions, ecological environment and socio-economic level of the different functional zones, we then proposed several development suggestions.

Previous scholars mainly explored the RECC of a single factor based on the prefecture-level cities and urban agglomeration, and failed to consider the complex mechanisms of the ecological environment, socio-economic and production activities on the RECC. In this paper, the RECC in the plain areas was evaluated on the basis of evaluation elements in major function-oriented zoning scheme. The RECC was shown to have strengthened the multi-element related research of social economy, resource endowment and ecological environment, etc. The paper also further bridged the theoretical research and practical application of the RECC, and enhanced the operability of research methods in terms of decision support aspect. Finally, the paper empirically analyzed the specific characteristics of each function zones in Fengxian County, which provides a reference for the optimization of territorial spatial pattern. The goals of our next phase of research will be dynamically monitoring changes of the RECC in the long term period, reasonably determining the overload threshold of the RECC in the study area, comparing the RECC to other research areas with different natural geographical environments, and further exploring the main factors affecting the RECC.

## Supporting information

S1 Fig Comprehensive evaluation of RECC. (ZIP)

S1 Table RECC at township level. (XLSX)
